# Machine learning applications for prediction of relapse in childhood acute lymphoblastic leukemia

**DOI:** 10.1038/s41598-017-07408-0

**Published:** 2017-08-07

**Authors:** Liyan Pan, Guangjian Liu, Fangqin Lin, Shuling Zhong, Huimin Xia, Xin Sun, Huiying Liang

**Affiliations:** 1 Institute of Pediatrics, Guangzhou Women and Children’s Medical Center, Guangzhou Medical University, Guangzhou, China; 2 Department of Hematology and Oncology, Guangzhou Women and Children’s Medical Center, Guangzhou Medical University, Guangzhou, China; 3 Department of Pediatric Surgery, Guangzhou Women and Children’s Medical Center, Guangzhou Medical University, Guangzhou, China

## Abstract

The prediction of relapse in childhood acute lymphoblastic leukemia (ALL) is a critical factor for successful treatment and follow-up planning. Our goal was to construct an ALL relapse prediction model based on machine learning algorithms. Monte Carlo cross-validation nested by 10-fold cross-validation was used to rank clinical variables on the randomly split training sets of 336 newly diagnosed ALL children, and a forward feature selection algorithm was employed to find the shortest list of most discriminatory variables. To enable an unbiased estimation of the prediction model to new patients, besides the split test sets of 150 patients, we introduced another independent data set of 84 patients to evaluate the model. The Random Forest model with 14 features achieved a cross-validation accuracy of 0.827 ± 0.031 on one set and an accuracy of 0.798 on the other, with the area under the curve of 0.902 ± 0.027 and 0.904, respectively. The model performed well across different risk-level groups, with the best accuracy of 0.829 in the standard-risk group. To our knowledge, this is the first study to use machine learning models to predict childhood ALL relapse based on medical data from Electronic Medical Record, which will further facilitate stratification treatments.

## Introduction

Acute lymphoblastic leukemia (ALL) is the most common malignant cancer among children^1^. Current risk-adapted treatments and supportive care have increased the survival rate to over 90% in the developed countries^2, 3^. However, approximately 20% of children who relapse have a poor prognosis, making ALL the leading cause of cancer mortality in pediatric disorders^4^. A major challenge in childhood ALL management is to classify patients into appropriate risk groups for better management. Stratifying chemotherapeutic treatment through the early recognition of relevant outcomes is critically important in order to mitigate poor disease courses in these patients^5^.

Previous group-level studies have identified many potential prognostic factors for childhood ALL, such as white blood cell (WBC) counts, age at diagnosis, response to prednisone and some gene fusions like BCR-ABL, TEL-AML1 and E2A-PBX1. Moreover, immunophenotype (T cell or B cell), percentage of lymphoblast in bone marrow (BM) on day 15 and day 33, level of minimal residual disease (MRD) may also help to identify the probability of relapse risk for patients at early therapy^3, 6, 7^. However, despite insight into various prognostic features, there is no clear consensus regarding how and which of these features should be combined for prediction. Clinicians still lack accurate tools to estimate a patient’s risk of ALL relapse in the early course of treatment.

Machine learning is a data-driven analytic approach that specializes in the integration of multiple risk factors into a predictive tool^8^. The application of different techniques for feature selection and classification in multidimensional heterogeneous data can provide promising tools for inference in medicine. Over the past several decades, such tools have become more and more popular for medical researchers, especially those in cancer research to predict the cancer susceptibility, recurrence and survivability^9, 10^. With the burgeoning availability of gene expression data and digitized phenotypic data, machine learning has been successfully used in nearly a dozen different kinds of cancers including leukemia^11–13^. However, to the best of our knowledge, there are no reliable machine learning tests that predict relapse in childhood ALL using existing medical data from Electronic Medical Record (EMR).

In this study, we applied, for the first time, state-of-the-art machine learning to sociodemographic, clinical, immunological and cytogenetic variables associated with ALL relapse to identify children with high relapse risk. In addition to the potential predictive values of our machine-learning methods, we also identified and ranked a limited set of prognostic factors in ALL patients.

## Results

### Patient characteristics

A total of 661 newly diagnosed ALL children were included in this study. After excluding 91 records with no or short follow-up time, there were 486 children receiving Guangdong – Acute Lymphoblastic Leukemia – 2008 (GD-ALL-2008, refers to the Chinese Children’s Leukemia Group –ALL – 2008 protocol^14^, detailed treatment therapy can be seen in Supplementary Table S1) and 84 children not. The follow-up time was at least 3.21 months, and the median was 31.87 months. During the follow-up period, 121 (21.2%) children relapsed after the first complete remission. The basic characteristics are listed in Table 1. The population distribution by gender was: 367 males and 203 females. Age at diagnosis ranged from 0.48 to 15.42 years old.

**Table 1:** Part of Patient Characteristics.

	Mean (SD)	Patients, N	Missing, N
Age (years)	4.72 (2.65)	570	0
Birth Weight (kg)	3.02 (0.43)	510	60
Hepatomegaly (cm)	2.49 (2.08)	557	13
Splenomegaly (cm)	2.04 (2.60)	557	13
White Blood Cell count (×10^9^/L)	40.23 (80.36)	570	0
Haemoglobin (g/L)	65.77 (23.12)	570	0
Platelet (×10^9^/L)	72.55 (80.20)	570	0
Peripheral Heterotypic cell (%)	0.30 (0.29)	565	5
Lactic Dehydrogenase (U/L)	910.11 (1631.24)	570	0
Ferroprotein (ng/mL)	540.61 (700.13)	570	0
Lymphoblast in Bone Morrow at diagnosis (%)	0.83 (0.12)	541	29
Lymphoblast in Bone Marrow on Day 15 (%)	0.10 (0.19)	570	0
Lymphoblast in Bone Marrow on Day 33 (%)	0.01 (0.02)	570	0
Minimal Residual Disease on day 15 (%)	8.07 (16.96)	233	337
Minimal Residual Disease on day 33 (%)	0.84 (3.69)	266	304
	**Categorical Value**	**Patients, N (%)**	**Missing, N**
Sex		570	0
	Male	367 (64.39)	
	Female	203 (35.61)	
Fever		553	17
	Yes	386 (69.80)	
	No	167 (30.20)	
Extramedullary Leukemia		570	0
	Yes	19 (3.33)	
	No	551 (96.67)	
Bone Invasion		417	153
	Negative	281 (67.39)	
	Positive	136 (32.61)	
Prednisone Response		570	0
	Poor	50 (8.77)	
	Good	520 (91.23)	
French-American-British		570	0
	L1	225 (39.47)	
	L2	285 (50.00)	
	L3	60 (10.53)	
BCR-ABL		570	0
	Negative	552 (96.84)	
	Positive	18 (3.16)	

### Model selection

Totally 103 clinical variables were left for model selection after the initial filtration. In Table 2, the accuracy, sensitivity, specificity, positive predictive value (PPV), negative predictive value (NPV) and area under the curve (AUC) of four machine learning algorithms with all the 103 features are reported. The tree-based algorithms, Random Forest (RF) and Decision Tree (DT), were clearly superior to Logistic Regression (LR) and Support Vector Machine (SVM), because of unbalanced low sensitivities versus high specificities of the latter methods. Furthermore, RF exhibited better prediction than DT in 4 of 6 measurements (accuracy: 0.831 vs. 0.791; specificity: 0.895 vs. 0.773; PPV: 0.880 vs. 0.781; AUC: 0.902 vs. 0.792). Therefore, of the four algorithms analyzed, the RF model seems to be most suitable for the relapse classification problem.

**Table 2:** Validated predictive performance of classifiers.

	Samples	Features	Accuracy	Sensitivity	Specificity	PPV	NPV	AUC	Ƙ*
Mean performances (±standard deviation) of four classifiers on 100 training sets with all features									
RF	150	103	0.831 ± 0.033	0.767 ± 0.058	0.895 ± 0.040	0.880 ± 0.041	0.795 ± 0.047	0.902 ± 0.030	—
SVM	150	103	0.719 ± 0.034	0.580 ± 0.069	0.859 ± 0.049	0.807 ± 0.050	0.673 ± 0.034	0.806 ± 0.055	0.553
LR	150	103	0.719 ± 0.037	0.601 ± 0.066	0.838 ± 0.046	0.788 ± 0.052	0.679 ± 0.051	0.802 ± 0.035	0.557
DT	150	103	0.791 ± 0.037	0.810 ± 0.055	0.773 ± 0.051	0.781 ± 0.045	0.804 ± 0.054	0.792 ± 0.037	0.596
Mean performances ( ± standard deviation) of RF on 100 test sets with 14 selected features									
RF	150	14	0.827 ± 0.031	0.756 ± 0.051	0.897 ± 0.041	0.882 ± 0.040	0.788 ± 0.044	0.902 ± 0.027	—
Performances of RF on independent 84 patients with 14 selected features									
RF	84	14	0.798	0.750	0.813	0.556	0.912	0.904	—

PPV, Positive Predictive Value; NPV, Negative Predictive Value; AUC, Area Under Curve; SVM, Support. Vector Machine; LR, Logistic Regression; DT, Decision Tree; RF, Random Forest.

### Features related to relapse

**Figure 1:**
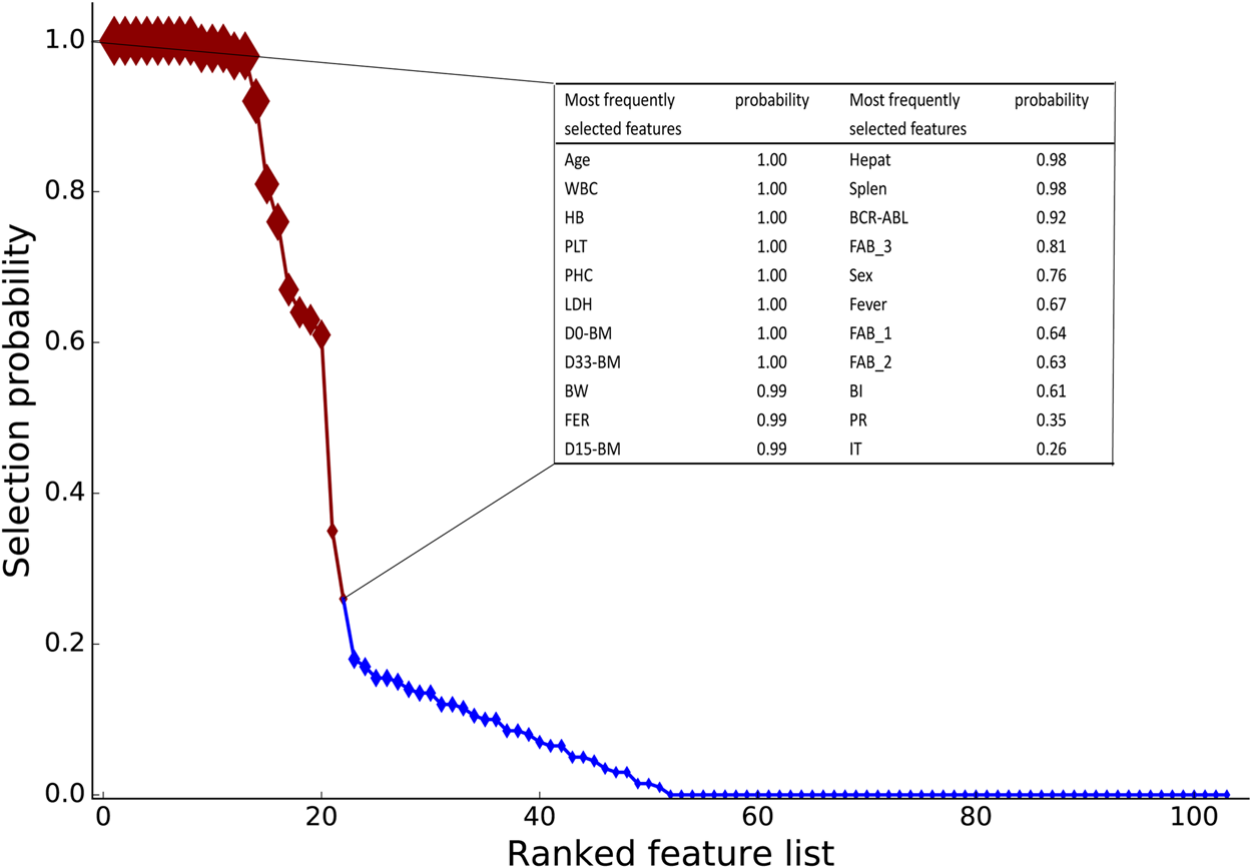
Composition of predictive variable sets selected by the nested cross-validation strategy. The features were ranked according to the selection probability measured across the 100 training sets. Variables with selection probability >20% were marked by red rhombuses and the details were shown in the upper right inset. WBC, White Blood Cell count; HB, Haemoglobin; PLT, Platelet; PHC, Peripheral Heterotypic cell; LDH, Lactic Dehydrogenase; D0-BM, Lymphoblast in Bone Morrow at diagnosis; D33-BM, Lymphoblast in Bone Morrow on Day 33; BW, Birth Weight; FER, Ferroprotein; D15-BM, Lymphoblast in Bone Morrow on Day 15; Hepat, Hepatomegaly; Splen, Splenomegaly.

The RF model was then applied for feature selection from training sets of 336 samples randomly split by 100-fold Monte Carlo cross-validation. As can be seen in Fig. 1, the top 22 features with selection frequency >20% were selected as potential predictors for the model development.

**Figure 2:**
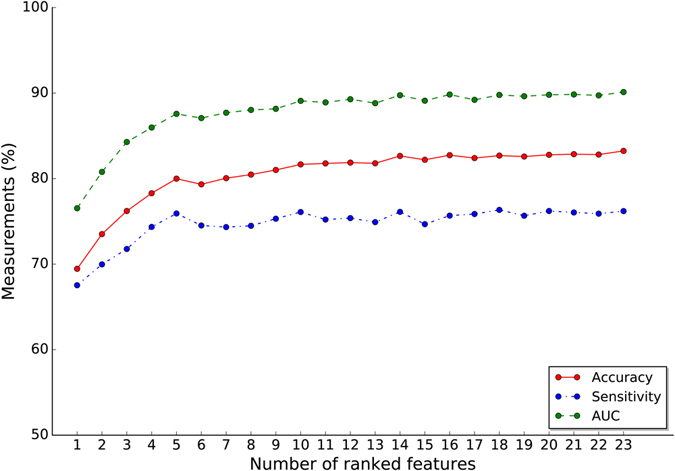
Classification accuracy, sensitivity and AUC of the random forest model along with the number of considered features. AUC, areas under curve.

To select the optimal number of features, we started to examine the predictive performance of the most prominent feature and identified the point at which there was no considerable gain in accuracy, sensitivity and AUC, when adding the feature of the next highest ranking one to the model. The maximum values reached for the three measurements defined the most discriminative features. The results of this forward feature selection process for the RF model were shown in Fig. 2. As expected, the three measurements increased gradually at first but then reversibly with the number of selected features, and reached their maximum values when considering 14 selected features as follows: Age, WBC, Haemoglobin (HB), Platelet (PLT), Peripheral Heterotypic cell (PHC), Lactic Dehydrogenase (LDH), Lymphoblast in Bone Morrow at diagnosis (D0−BM), Lymphoblast in Bone Marrow on Day 33 (D33−BM), Birth Weight (BW), Ferroprotein (FER), Lymphoblast in Bone Marrow on Day 15 (D15−BM), Hepatomegaly (Hepat), Splenomegaly (Splen), and BCR-ABL.

Nowadays, MRD is found to be perhaps the most important predictor of ALL relapse. Within the GD-ALL-2008 protocol, MRD analysis was performed as an add-on study, resulting in a relatively high missing rate of MRD in our samples (58.23% for MRD on day 15, 283 out of 486; 51.44% for MRD on day 33, 236 out of 486). To investigate how MRD is related to our model, we included MRDs (D15-MRD, MRD on Day 15; D33-MRD, MRD on Day 33) into the selected feature set, trained and evaluated the RF model on the 193 samples with both MRDs on day 15 and 33. From the results (Supplementary Table S2), the addition of MRDs as predictors indeed improved the performance of the model. However, the improvement was not significant, with the accuracy being increased only from 0.821 to 0.837, the sensitivity being increased from 0.690 to 0.731, meanwhile the AUC being increased from 0.884 to 0.903. This might partly due to the small sample size or low stability of the flow cytometry technology used in MRD detection. Therefore, MRDs were not included in the optimal feature set in this study.

### Prediction of relapse

Two independent test sets were used to evaluate the prediction power of the RF model with 14 selected features. First, test of 150 samples in the 100 test sets randomly split by Monte Carlo cross-validation on the RF classifiers resulted in an accuracy of 0.827 ± 0.031, a sensitivity of 0.756 ± 0.051, a specificity of 0.897 ± 0.041, and an AUC of 0.902 ± 0.027. These measurements are thought to be truer estimate of the classification performance than obtained from 10-fold cross-validation during training. Second, to further verify the robustness of our model, we extracted the clinical data of 84 ALL children who did not receive GD-ALL-2008 as the second independent test set. As expected, the RF model also performed well on this data set, with an accuracy of 0.798, a sensitivity of 0.750, a specificity of 0.813, and an AUC of 0.904. This result suggests that the RF model has a good generalization ability in the prediction of childhood ALL relapse.

### Performance of the model in different risk-level groups

Clinically used risk allocation schemas have been changing with the great improvement of knowledge of the ALL biology over the past few decades. However, there are still many standard-risk patients who relapsed and many high-risk patients who received overtreatment. So it is in this study, where more than half of the relapsed children (15 out of 28) belong to these two groups (Table 3), suggesting the current risk stratification is fairly non-specific.

**Table 3:** Prediction performances on stratified risk groups of the 84 patients.

Risk group	Total (N)	Relapsed (N)	Accuracy	Sensitivity	Specificity
Standard-Risk	26	3	0.829	0.579	0.880
Intermediate-Risk	30	12	0.699	0.691	0.705
High-Risk	28	13	0.821	0.855	0.739

We evaluated the performance of the RF model in different risk-level groups in the second test set with 84 children to verify its applicability. As expected, the model performed well in all the groups, with the accuracy ranging from 0.699 for the intermediate-risk group to 0.829 for the standard-risk group (Table 3). Of 3 relapses in the standard-risk group, the model made 2 correct predictions; of 12 relapses in the intermediate-risk group, the model made 9 correct predictions; of 13 relapses in the high-risk group, the model made 11 correct predictions. In spite of these fairly satisfactory results, it should be noted that the performances are not equal across different groups, with the sensitivity changing from 0.579 to 0.691, and to 0.855, while the corresponding specificity changing from 0.880 to 0.705, and to 0.739, as the risk level increased.

## Discussion

The relapse of childhood ALL after treatment increases the risk of death. There is a great need for the ability to predict the treatment outcome to be used in the treatment planning. Currently, no screening tool exists in clinical that is expressly designed to distinguish relapse with high accuracy. In this study, we predicted the relapse in pediatric ALL in the context of machine learning algorithms. Among the various machine learning algorithms, RF proved superior to the other algorithms utilized herein. The algorithm achieved good performances on both independent test sets. These results provide the first evidence that a machine learning algorithm can discriminate between children with and without relapse using clinical data from EMR.

So far, several relapse prediction models using machine learning methods have been reported for pediatric ALL^15–17^. These models were based on gene expression data or combined MRD and IKZF1 (Ikaros zinc finger-1 gene status), and achieved accuracies ranging from 0.735 to 0.790. Our RF model exactly is a little better than the existing models, with a cross-validation accuracy of 0.827 ± 0.031 on one test set and an accuracy of 0.798 on the other. The AUC of our model can reach as high as 0.904. On the other hand, being different from the gene data, the features used in our model can be easily obtained with low cost, thus providing the chance for the model to be used in a clinical setting as early as possible.

Feature selection usually plays a significant role in machine learning, as irrelevant attributes may lead to low accuracy, less interpreted and over-fitting results in classification analysis^18^. Furthermore, it is also valuable for clinicians to know the most predictive variables for treatment outcome, as they can make personalized treatment plan taking these variables into account. A large number of potential relapse predictors have been outlined by previous studies^6^. However, the choice of variables by experts and literatures available may introduce bias^19^. To understand better and objectively which baseline characteristics might be the strongest predictors of relapse at the individual patient level, we adopted a feature selection strategy which combines 100-fold Monte Carlo cross-validation nested with 10-fold cross-validation to repeatedly screen potential variables. All the variables were ranked according to the probability that each variable would be selected, which might serve as a better indicator for a variable’s importance. In fact, most of the top 22 features with selection frequency >20% have been indicated or used as prognostic factors for childhood ALL^7, 20–24^, supporting the validity of this strategy.

Our results showed that the tree-based algorithms, RF and DT, were well-suited to the classification task at hand. This is expected, considering the simplistic nature of the algorithms, and the safeguards they have against over-fitting. The RF model yielded sufficient and best classification performance with only the top 14 features. Almost all these features except D33-BM (the 8^th^ one) were measured at first diagnosis. With the exception of one feature (BI), each of our feature was available in >90% of patients, even the majority being present in 100% of the patients. Data used in the study were from medical center’s EMR without additional clinical assessments. It means that our prediction algorithm can be performed as early as possible even at the time of the first risk stratification evaluation and easily integrated into EMR to identify children at elevated or decreased relapse risk. Treatment intensity could thus be adjusted accordingly.

Clinically, ALL children were stratified into 3 groups based on risk of relapse, which was predicted by a combination of clinical, cytogenetic, and morphological variables^14, 25–27^. Although great improvement has been achieved in the ALL treatment with the risk-stratified therapy, most relapses arise in intermediate-risk and standard-risk groups^25–27^. Thus, more work is needed to identify the children at high risk for relapse. Besides looking for new prognostic factors, the prediction models developed here provides another way to predict relapse with the combination of currently used factors. Our RF algorithm can be applied to distinguish relapses and non-relapses across all the three risk-level groups, with the best accuracy of 0.829 obtained in the standard-risk group. However, the algorithm was less sensitive to classifying relapse among children of the standard-risk group, while less specific to that of the intermediate-risk group. It is possibly due to a highly imbalanced ratio of relapsed to non-relapsed children in the data set.

One of the most promising aspects of the RF model is the hope that it will identify patients at high relapse risk, thus giving them intensive or novel treatment strategies, which might significantly influence the chances for cure. Second, for patients with favorable prognosis, the early identification by the model might be of help to get benefit from treatment reduction, complications decline, and medical costs decrease. Third, the feature sets selected in the model should provide critical insights into the underlying mechanisms that contribute to relapse. We anticipate that future studies may reveal whether the ALL treatment would benefit from the use of the model.

Despite of our promising results, some limitations of the present exploratory study should be considered. First, the sample size used for model development and evaluation is relatively small as we employed only one center’s data. Therefore, in future studies, more data such as that acquired through a multicenter research should be used to validate the generalization of the prediction models. Second, in order to solve the data imbalance problem, we used the well-known Synthetic Minority Over-sampling Technique (SMOTE) over-sampling method to generate new synthetic samples from the relapsed children, which might influence the models’ performance. Third, the strong predictors, MRD and some gene fusions, were not included in our variable set due to unacceptable data missing rate. Adding these variables may enhance predictive power and aid treatment personalization. However, on the other hand, the problems of cost, complexity and reproducibility in clinical use of these variables^28, 29^ might hinder the applicability of the prediction model unless these hurdles disappear as technologies advance.

In conclusion, we show that machine learning algorithms based on already obtained clinical and laboratory variables can predict relapse of pediatric ALL. While the current model needs to be further improved before clinical use, the significant predictive value of our RF classification approach might have potential to provide useful information for the clinical practice of stratifying chemotherapeutic treatment, thus encouraging a computer-aided treatment perspective.

## Materials and Methods

### Population and outcome

**Figure 3:**
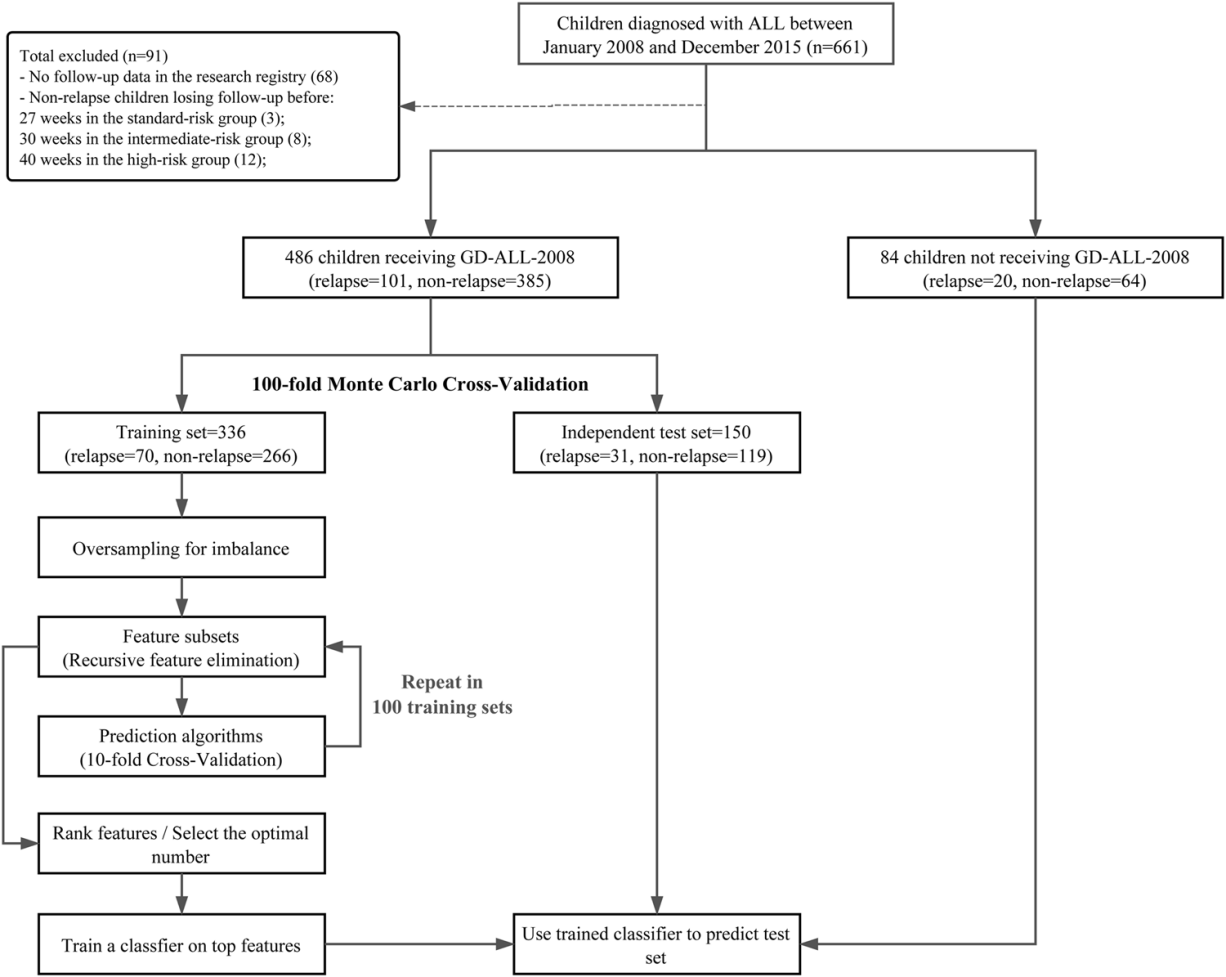
Flow chart of data collection, data preprocessing, feature selection and model development. The overall data set was made up of 570 patients and 121 out of them were relapsed.

Between January 2008 and December 2015, 661 children aged <16 years, who were newly diagnosed with ALL and achieved complete remission after the induction therapy, were enrolled into the retrospective study at Guangzhou Women and Children’s Medical Center, Guangzhou, China. Most of patients were treated with GD-ALL-2008 except 84 children who received other treatment protocols. The 661 children were stratified into 3 risk groups (standard-risk; intermediate-risk; high-risk) based on variables at first diagnosis and early therapy response, such as age, WBC in peripheral blood, immunophenotype, gene fusion, karyotype, prednisone response, day 15 and day 33 BM remission status, MRD on day 33, central nervous system (CNS) status and so on ref. 14. Details of risk stratification and chemotherapy stages of GD-ALL-2008 protocol are demonstrated in Supplementary Table S1. We excluded 68 children because of unidentified and/or incomplete follow-up. We also excluded 23 children who lost follow-up during the maintenance period before 27 weeks in the standard-risk group (3), 30 weeks in the median-risk group (8) and 40 weeks in the high-risk group (12) from the non-relapse children cohort according to the treatment protocol. Thus the resulting 570 ALL children were included in the study population (Fig. 3). Relapse in ALL includes three situations: any of bone marrow (BM) relapse defined as the reappearance of leukemic cells in BM (>25% blasts), central nervous system leukemia relapse defined as more than 5% blasts in the cerebrospinal fluid, and testis leukemia that was diagnosed clinically and confirmed with ultrasonography.

This study was reviewed and approved by the institutional ethical committee board of Guangzhou Women and Children’s Medical Center (No. GO-2016–017), conducted in accordance with the ethical guidelines of the Declaration of Helsinki of the World Medical Association. The requirement to obtain informed consent was waived because of the retrospective nature of the study. Data used in this study were anonymous and no identifiable personal data of the patients were available for the analysis.

### Predictor variables

In the study, we adopted a data-driven, multivariate analysis strategy and did not preselect a small set of predictor variables. Instead, all the clinically relevant biochemical, morphological, immunological and cytogenetic variables measured at first diagnosis or at complete remission (day 33) were collected from EMR. Polytomous variables should be converted into three or more binary attributes. For example, French–American–British (FAB) morphological classification was divided to three binary variables, FAB_1, FAB_2, FAB_3, representing a kind of cell morphology, L1, L2 and L3, respectively. Genetic variables such as TEL-AML1 had a low sampling frequency due to the lack of laboratory equipments before the year 2012. Here we removed potentially difficult-to-obtain variables with missing rates more than 50%, such as MRD and fusion genes TEL-AML1 and E2A-PBX1, leaving 103 features for analysis.

### Machine learning strategies

The aim of the study is to select prognostic factors related to relapse and predict the need of intensive treatment in case of relapse in ahead with machine learning models. The main process can be divided into four steps: (1) data preprocessing, the process of resolving problems of missing data values and data imbalance, (2) model selection, the process of selecting a suitable classification algorithm, (3) feature selection, the process of selecting a subset of relevant features to be used for classification, and (4) model development and validation, the process of using the selected features to separate the two groups of subjects (relapse vs. non-relapse). Figure 3 shows the flowchart of our work.

### Data preprocessing

There are in total 570 ALL children in the study, of which 84 children not receiving GD-ALL-2008 treatment were excluded from model development and cross-validation analysis, serving as an independent test set. For the remaining 486 children, a Monte Carlo cross-validation^30, 31^, was established to eliminate the bias in the evaluation that may occur due to sampling effect. Concretely, cases and controls were randomly split into two experimental datasets by 100 times, a training set with 70% of patients (including 70 cases and 266 controls) and a test set with 30% of patients (consisting of 31 cases and 119 controls). The ratio between cases and controls was maintained in the testing set to preserve the generalizability of the study’s findings.

Good quality of data can improve the performance in feature selection and prediction, which indicates that data preprocessing is a necessary step before machine learning. Two main problems existing in our medical data sets are as follows:

1. *Missing values*: Some variables have high missing rates in the original data set although we have removed those variables with >50% missing rates. In order to get a higher quality of dataset, imputation and normalization were done before data was input to the model. The missing values were filled with median, mean or categorical values according to the meaning of corresponding characteristics (i.e., BW and clinical attributes were filled with median values and Fever was filled with “No” for the reason that patients who fevered would be recorded with specific temperature values).
2. *Imbalance*: Our data set includes 121 positive samples (relapse) and 449 negative ones (non-relapse). In classification models, learning algorithm tends to have high accuracy over the majority, but put less weights over the minority, thus causing an imbalance problem which may impact the performance of classifiers seriously^32^. Here, SMOTE, the over-sampling method^33^ was used to generate synthetic samples for the minority class and thus balanced the positives and negatives in the training sets.

### Model selection

For relapse prediction, four classification algorithms were applied and evaluated: SVM, LR, DT and RF. The main reasons for choosing these algorithms are due to their low computational cost, clear interpretation, and the possibility of being applied to both discrete and continuous variables. SVM with linear kernel fits samples well if the data size is small. LR is popular in prediction due to its simplicity and fast computation speed. Unlike other black box classifiers, DT is convenient to be interpreted with tree plot. RF is an ensemble classifier based on many simple decision trees. Classifiers were trained and evaluated by a 10-fold cross-validation on each of the 100 training sets. The resulting final performance of each model was obtained by averaging over the 100 evaluations.

### Feature selection

Feature selection is crucial in improving a model’s efficiency and performance^34^. Similar to the process of model selection, only the training set was used for the feature selection. An optimal feature set was selected for each training set by using recursive feature elimination^35^, together with the classification algorithm selected above. Features were ranked according to the times of being selected in the 100 training sets. In pursuit of everyday clinical applicability, variables with selection frequency >20% were chosen as potential predictors for relapse and are detailed in Table 1.

As the most discriminatory variables have been determined and ranked, our aim is to determine the feature set with least variables but highest predictive accuracy. The algorithm to find the minimum-size list of features is the Forward Feature Selection, trying to add features that improve classifier’s performance from the feature set, which is similar to the Backward Feature Elimination^36^.

The algorithm of Forward Feature Selection works as follows:

1. Beginning by the head of the ranked list of predictor variables, the algorithm iteratively generates increasingly longer lists by adding one variable at a time, builds a new model and calculates its classification accuracy.
2. The list with the minimum size and optimum accuracy is therefore selected.

### Model development and validation

As both the algorithm and the minimum-size list of features have been determined, the classifier was trained on the training set (336 patients), using the training set feature values as input. After training, the classifier was asked to predict the relapse of the test set (150 patients). This process was repeated 100 times with different training and test sets, and the resulting final performance was obtained by averaging over the 100 evaluations. Then, using the total 486 children receiving GD-ALL-2008 treatment, the model was retrained and applied to the 84 patients not receiving GD-ALL-2008 for evaluation. All models were developed in Python using the package Scikit-learn^37^.

### Model Assessment

Measurements are necessary in selecting a suitable model to predict unseen samples among models. In our study, accuracy, sensitivity, specificity, positive predictive value (PPV) and negative predictive value (NPV) were calculated from the confusion matrix to quantify the performance of the proposed classification algorithms. Confusion matrix is a matrix about comparison between prediction and actuality, containing rows and columns. Each column represents the predicted results while each row represents the actual ones. In binary classification, the dimension of confusion matrix is 2 × 2 and is composed by: True Positives (TP), True Negatives (TN), False Positives (FP) and False Negatives (FN). TPs are positive samples correctly predicted as positives and TNs are negative samples correctly predicted as negatives. FPs are negative samples incorrectly identified as positives and FNs are positive samples incorrectly identified as negatives. Conceptions about measurements used in the study are described as follows:

1. Accuracy is the proportion of correctly classified samples among total number of samples.
  $$accuracy=\frac{TP+TN}{TP+FP+TN+FN}$$
2. Sensitivity is also called the true positive rate or recall. It is the fraction of positives that are correctly retrieved. In medical data, we expect the predictors to get high sensitivity as possible so that disorders in patients could be discovered in time. Sensitivity is related to which disorders are not overlooked.
  $$sensitivity=\frac{TP}{TP+FN}$$
3. Specificity measures the proportion of negative samples correctly identified among all the samples that are identified as negatives. It is related to which disorders are not mistaken.
  $$specificity=\frac{TN}{TN+FP}$$
4. PPV measures the proportions of positive results in statistics and diagnostic tests that are true positive results.
  $$PPV=\frac{TP}{TP+FP}$$
5. NPV measures the proportions of negative results in statistics and diagnostic tests that are negative positive results.
  $$NPV=\frac{TN}{TN+FN}$$
6. Receiver operating characteristic (ROC) curve and AUC

ROC curve is commonly used to illustrate the performance of a classifier through showing the tradeoff between the sensitivity and specificity. AUC is another effective indicator for assessing the prediction model’s discrimination. The AUC of a perfect model can reach to 1, while that of a random guessing curve which is a diagonal line in the graph would equal 0.5.

## Electronic supplementary material

Supplementary Information
