# Supplementary material for: Machine learning applications for prediction of relapse in childhood acute lymphoblastic leukemia
Source: Sci Rep. 2017 Aug 7;7:7402. doi: 10.1038/s41598-017-07408-0 (PMC5547099; doi:10.1038/s41598-017-07408-0)
Supplement: Supplementary file 1 — Supplementary Information [file 41598_2017_7408_MOESM1_ESM.pdf]

Machine learning applications for prediction of relapse in childhood acute lymphoblastic leukemia

Liyan Pan[1,†], Guangjian Liu[1,†], Fangqin Lin[1], Shuling Zhong[2], Huimin Xia[3], Xin Sun[2,*], Huiying Liang[1,*]

[1]Institute of Pediatrics, Guangzhou Women and Children's Medical Center, Guangzhou Medical University, Guangzhou, China;

[2]Department of Hematology and Oncology, Guangzhou Women and Children's Medical Center, Guangzhou Medical University, Guangzhou, China;

[3]Department of Pediatric Surgery, Guangzhou Women and Children's Medical Center, Guangzhou Medical University, Guangzhou, China;

[†] Both authors contribute equally to this paper

[*]Corresponding Authors:

Huiying Liang, Institute of Pediatrics, Guangzhou Women and Children's Medical Center, Guangzhou Medical University, Guangzhou, 510623, email: lianghuiying@hotmail.com

Xin Sun, Department of Hematology and Oncology, Guangzhou Women and Children's Medical Center, Guangzhou Medical University, Guangzhou, 510623, email: doctorsunxin@hotmail.com

Table S1. Risk stratification criteria and chemotherapy courses of GD-ALL-2008 protocol. SR, Standard-Risk; IR, Intermediate-Risk; HR, High-Risk.

Risk Group	Risk Stratification	Induction Period(weeks)	Consolidation Period(weeks)	Delayed Induction Period(weeks)	Maintenance Period(weeks)
SR	All the factors: (1) Prednisone good response; (2) 1 year≤Age<6 years; (3) WBC counts at initial diagnosis < 20×10^9/L; (4) D15-BM <5% or 5%-25%, D33-BM < 5%; (5) B or B-precursor ALL; (6) BCR-ABL fusion gene negative or no t(9;22) chromosomal translocation; (7) no MLL gene rearrangement; (8) No extramedullary ALL at initial diagnosis.	0-7	9-17	19-26	28-102
IR	At least one condition: 1.(1) Prednisone good response; (2) Age <1 year or Age ≥6 years; (3) WBC counts at initial diagnosis ≥ 20×10^9/L; (4) D15-BM <5% or 5%-25%, D33-BM <5%; (5) T-ALL; 2.Conformed to SR standards, D15-BM >25%; 3. Conformed to SR standards, D15-BM <5% or 5%-25%, D33-BM <5%.	0-10	12-20	22-29	31-106
HR	At least one condition: 1. Conformed to IR standards, D15-BM >25%; 2. Prednisone poor response; 3. D33-BM 5%-25% or >25%; 4. BCR-ABL fusion gene positive or t(9;22) chromosomal translocation; 5. MLL-AF4 fusion gene positive or MLL gene rearrangement; 6. with testicular leukemia or mediastinal tumor at initial diagnosis and reduction rate of lesion size <70% on day 33 after induction.	0-10	12-28	32-39	41-143/171

Table S2. Validated predictive performance of classifiers. PPV, Positive Predictive Value; NPV, Negative Predictive Value; AUC, Area Under Curve; SVM, Support Vector Machine; LR, Logistic Regression; DT, Decision Tree; RF, Random Forest.

	**Samples**	**Features**	**Accuracy**	**Sensitivity**	**Specificity**	**AUC**	**PPV**	**NPV**
Mean performances (±standard deviation) of RF on 100 training sets without MRDs								
RF	193	14	0.821±0.047	0.690±0.095	0.909±0.052	0.884±0.044	0.833±0.088	0.817±0.057
Mean performances (±standard deviation) of RF on 100 training sets with MRDs								
RF	193	16	0.837±0.052	0.731±0.100	0.909±0.051	0.903±0.041	0.841±0.083	0.837±0.064
